# Anticipatory grief and experience of providing at-home palliative care among informal caregivers of spouses in Croatia: a qualitative study

**DOI:** 10.1186/s12904-022-01093-1

**Published:** 2022-11-18

**Authors:** Jelena Bilić, Lea Skokandić, Livia Puljak

**Affiliations:** 1 Center for Evidence-Based Medicine, Catholic University of Croatia, Ilica 242, 10000 Zagreb, Croatia; 2 Faculty of Law, Social Work Study Centre, University of Zagreb, Ulica Vladimira Nazora 51, 10000 Zagreb, Croatia

**Keywords:** Palliative care, Anticipatory grief, Informal caregivers, Qualitative study

## Abstract

**Background:**

In palliative care, caring for spouses suffering from incurable diseases can provoke a range of reactions in informal caregivers that are part of the grieving process, as well as other reactions and ways of coping with a current role, which is often challenging. Anticipatory grief occurs before death and is often present in people who face the eventual loss of a loved one or their own death. This study aimed to gain insight into the anticipatory grief of informal caregivers who are providing at-home palliative care for their ill spouse. Our research questions focus on investigating the meanings caregivers ascribe to the experience of providing palliative care and the impending loss of a spouse.

**Methods:**

A qualitative study was conducted in Zagreb, Croatia, from April to June of 2021. Eight participants took part in the study. Participants in the study were informal caregivers of a spouse suffering from an incurable, terminal disease that receives at-home palliative care. Data were collected through semi-structured face-to-face interviews. Transcripts were analyzed by interpretive phenomenological analysis.

**Results:**

The analysis provided several meanings that represent caregivers’ experiences and coping strategies. The caregivers bravely face the challenges of “living with an illness” by maintaining optimism, strong cohesion with their partner and a sense of joint destiny. They tend to repress their own personal needs and feelings while carrying the burden of care. Caregivers tend to stay positive and focus on living in the present by taking an active role in providing care for the ill spouse and family.

**Conclusions:**

Anticipatory grief presents emotional, cognitive, and spiritual challenges to spouse caregivers in palliative care. The contribution of this study was to gain insight into the meaning that caregivers ascribe to the experience and challenges they face while providing everyday care for their ill spouse. Confirming prior results, the experiences are generally similar to all caregivers, pointing to the need for substantial improvement in the quality of the support and help from the healthcare workers and other experts who provide palliative care and support for the patients’ family members.

**Supplementary Information:**

The online version contains supplementary material available at 10.1186/s12904-022-01093-1.

## Background

Anticipatory grief was defined as “any grief occurring prior to a loss, as distinguished from the grief which occurs at or after a loss” [1]. The term was initially introduced in the context of the Second World War, as it was observed that relatives of the soldiers passed through the phases of grief [2]. The concept of anticipatory grief is now better understood in the context of many situations and various types of losses. The term is sometimes interchangeably used with other descriptors in the literature, such as pre-death grief, or preparatory grief or anticipatory mourning. Anticipatory grief can be experienced both by a dying person and their loved ones. Symptoms of such grief can be physical, emotional, cognitive and/or spiritual. Compared with the “conventional grief” after death, it was found that anticipatory grief was characterized by higher intensities of anger, loss of emotional control, and atypical grief [3].

Regarding the concept itself, Fulton expressed serious reservations regarding the theoretical or practical heuristic value of the concepts, ‘‘anticipatory grief’’ and ‘‘anticipatory mourning’’ [4]. A systematic review of Kjaergaard Nielsen et al. investigated the main issues related to anticipatory grief and preparedness for the death, including definitions, measurement tools, and potential effects on caregiver outcome. They included 34 studies, of which the majority were longitudinal. They reported that anticipatory grief was captured in the definition “pre-loss grief.” High grief levels and low preparedness levels during caregiving were associated with poor bereavement outcome, such as complicated grief. The authors concluded that the hypothesis that grief work before the loss would alleviate bereavement outcome was not confirmed. Thus, the authors questioned the concept of anticipatory grief. Their suggestion was that the terminology of grief symptoms before the death of a patient might benefit from a shift away from the use of anticipatory grief and towards loss and grief during caregiving. Nevertheless, the authors highlighted the need for specific interventions for caregivers’ pre-loss grief and further development of preparedness interventions to improve and target support for the caregivers in the future [5].

It has also been suggested that the challenges with using the conceptual idea of anticipatory grief include a narrow focus of the traditional view of grief. Evans suggested that this concept includes a limited view of the “death event” as the only loss, and that all other losses associated with the terminal disease need to be considered as variables that may determine the anticipatory grief experience [6].

Experiences typical of anticipatory grief were described in informal caregivers of people with incurable diseases [7]. An informal caregiver is any individual, such as a family member or a friend, who provides regular, ongoing assistance to another individual without payment for the care given. It has been described that such informal caregivers are increasingly providing care to those in need of palliative care. However, the majority of informal caregivers do not have prior knowledge related to the provision of care. Their role as informal caregivers is very burdensome, including the need to face the patient's imminent death [8].

It has been suggested that a palliative care model that includes the family could help address complex psychological needs of informal caregivers providing palliative care and that a better understanding of the concept of anticipatory grief as experienced by informal caregivers would be necessary for formulating a palliative care model encompassing all needs [9].

In Croatia, organized palliative care is in its inception and, to our best knowledge, there are no published studies about experiences of informal family caregivers providing palliative care. Thus, it is unknown how informal family caregivers of terminal patients cope when they need to provide palliative care, what kind of help they would need from healthcare workers, and whether they experience anticipatory grief. Better insight into those experiences might help healthcare workers to better help patients and their families.

This study aimed to gain insight into the anticipatory grief of informal caregivers who are providing at-home palliative care for their ill spouses. Our research questions focus on the investigation of the meanings caregivers ascribe to the experience of providing palliative care and the impending loss of a spouse.

## Methods

### Ethics

The study protocol was approved by the Ethics Committee of the Catholic University of Croatia and the Ethics Committee of the Health Center Zagreb-East, whose patients cared for spouses who participated in this research. The study was performed according to the Declaration of Helsinki. The methods were carried out in accordance with the relevant guidelines and regulations, including the institutional ethics code.

The participants signed informed consent to participate in the research after receiving verbal and written information about the study’s aims. Thus, all participants gave their written informed consent for participation in the study. The complete text of information about the study can be found in Additional file 1: Appendix 1. The text of the informed consent is in Additional file 1: Appendix 2. The form was signed in two copies, one of which was kept by the participant and the other by the researcher.

Due to sensitivity of topics, i.e. conversation about the imminent death of the spouse, the participants were given information about the contact of an expert/institution that they could contact in case of adverse psychological consequences due to participation in the research (La Verna Association Counseling).

### Study design

A qualitative study was conducted using the semi-structured interview method. A qualitative, phenomenological approach was applied, which enabled insight into the in-depth experiences of anticipated grief in caregivers. The research protocol was written according to the guidelines for reporting on qualitative studies, *Consolidated Criteria for Reporting Qualitative Studies*, COREQ) [10].

### Setting

The study was conducted from April to June 2021 in the city of Zagreb, Croatia. The participants were invited to propose a location for the interview.

### The research team and reflexivity

Personal characteristics of the researcher: All interviews were conducted by one person (JB, the first author), a female nurse with a bachelor’s degree, finalizing the Master’s studies in nursing, with 3 years of work experience in palliative care. The researcher had no previous experience in conducting qualitative research. She was educated for research purposes and conducted interviews under the supervision of the two mentors. LS and LP have mentored and prepared JB for conducting the interviews because JB has extensive experience working with the patients in need of palliative care and their carers. JB and LS completed three specialist postgraduate courses in palliative care, the only formal palliative care education in Croatia. LS also completed 320 h of practical work at the children’s oncology department within her palliative care training. The mentors, LS and LP, are university professors with expertise in research methodology and experience in teaching about and conducting qualitative studies from the field of social and biomedical sciences [11–13].

Relationship with participants: The researcher who conducted interviews did not have any previous relationship with the participants, and neither did the other co-authors. Participants did not previously know the researchers. Participants were told that interviews were conducted for research purposes.

### Participants

Participants were informal caregivers of a spouse suffering from an incurable disease with a certain fatal outcome. We purposefully selected individuals providing informal care for their spouse who is in the terminal phase of a disease and who were aware that the active treatment of the disease was terminated and that this means that their spouse is facing certain death. A dying spouse had to receive at-home palliative care for at least one month during 2021 by a mobile palliative team in the area of the city of Zagreb.

The participants were selected by the non-probabilistic method of purposeful sampling. They were contacted by telephone based on the pre-assessment of potential eligibility by the Mobile Palliative Team of the Zagreb-East Health Center.

### Data collection

Data collection was conducted by face-to-face semi-structured interviews in the Croatian language using a pre-determined list of questions. The participants were encouraged during the interview to express additional relevant information that was not foreseen by those questions. Audio-recording of conversations was made using a mobile phone and then transcribed with the removal of all data that could compromise the anonymity of the respondents. The duration of the interviews was between 30–40 min. The interviews were not repeated. The participants were not provided with transcripts for review.

Three pilot interviews were conducted; their purpose was to test the suitability of the questions from the study protocol. After that, the team held a discussion, and the need to revise one question was identified. The second research question referred to professional help and support that caregivers consider important during the provision of palliative care to a spouse. In the pilot interviews, answers to these questions were very brief, which led to a conclusion in the research team that the participants may not be sufficiently informed about how experts could help them and what forms of help are available. Thus, this question was expanded in a way to put more emphasis on their experiences, i.e. What do they do? How are they?, What should they do?. A complete list of questions used in the interviews is in Additional file 1: Appendix 3. The participants who participated in the pilot interviews were included in the main sample.

### Data analysis

The data were analyzed using the interpretative phenomenological analysis (IPA) [14]. The analysis included several iterative phases. The first author (JB) prepared empirical material for analysis (data collection and transcription), while analysis was done in collaboration with other two team members. The names of all respondents in the transcripts of the interviews were coded; only the interviewer knew their identity.

Each transcript was examined separately and read several times. While reading, the authors divided the text into units according to specific meaning and assigned comments and reflections in the margins. Some comments were paraphrasing or summarizing the participants’ responses, while the others were pointing toward preliminary interpretations or emerging *themes*. After listing and clustering similar themes in relation to the aim of the study, these similar themes formed a basis for the *meanings*, while keeping the link to the primary source material. When this interpretative process was done for the first case, the same procedure was done with others. After several iterations, the final list of themes from the first case was used as an orientation for the subsequent analysis, respecting convergences and divergences in the meaning of participants’ experiences in the remaining data. Finally, we constructed a table of themes that pointed toward several core meanings that were evident from all the cases taken together. In interpretation, every *meaning* was described and interpreted using themes that best reflect and capture the content of each *meaning*.

The use of the IPA method in this study aims to achieve a comprehensive understanding of lived experiences of anticipatory grief in which individuals try to interpret and understand what they feel from their perspective [14]. Thus, the results in this study represent the topics, i.e. the *meanings* to which the participants’ answers refer, and they relate to the research question. Multiple themes were repeated by different participants, while some were unique to individual participants because they reflected their personal specific care experience, which differed from others’.

Complete sentences from the original transcripts of the interviews were used in the reporting to convey the original thoughts of the participants. Quotations are listed under the code of the participants. Respondents were not asked for feedback on the final results.

## Results

### Participants

A total of 8 caregivers participated in the study. Five individuals agreed to participate, but due to the death of the caregiver shortly after they provided informed consent, they no longer met the inclusion criteria. Two individuals refused to participate in the study, stating that the condition of the spouses requires increased care and, therefore, they are unable to leave them even for the time required to conduct the interview.

Six interviews were conducted in the participants 'homes, while two were conducted in a public place (Caffe bar), based on the participants’ wishes. In 5 cases, the caregivers agreed that the spouses were informed about the content of the interview, while the other 3 wanted the spouses not to be informed about it. Only the respondent and the graduate were present during the interview. During the conversation, notes were kept, which referred to the non-verbal signs that the respondents showed (tears, change of facial expression, sighs, etc.).

The study involved seven women and one man, aged 38 to 75 years. Respondents were informal caregivers of their spouses from 6 months to 4 years. The participants’ characteristics and codes are shown in Table 1.

**Table 1:** Participants’ characteristics and codes

Code	Participant’s sex	Participant’s age (years)	Duration of care for the spouse	Spouse’s sex	Spouse’s age (years)	Spouse’s diagnosis
P1	Woman	69	6 months	Man	74	Lung cancer
P2	Woman	74	2 years	Man	77	Status post-cardio-vascular insult, prostate adenoma, dementia
P3	Man	63	3 months	Woman	63	Lung cancer
P4	Woman	66	4 years	Man	69	Kidney cancer
P5	Woman	53	5 months	Man	59	Tongue cancer
P6	Woman	75	2 years	Man	83	Prostate cancer; chronic obstructive pulmonary disease
P7	Woman	38	1 year	Man	40	Spinal cord cancer
P8	Woman	72	4 years	Man	82	Lung cancer

### What is the meaning that the informal caregivers ascribe to the impending loss of a spouse and to the experience of at-home palliative care for their spouse?

*“At one point, you feel that maybe like a panic attack.......what to do next.....you say to yourself, look, I have to be strong, you know what, I always remember that movie, it sounds funny...that “Gone with a wind” it's an old movie ..it comes to my mind many times when I'm having a hard time, she turns around and says “Tomorrow is a new day”. Tomorrow is a new day, I have to be strong... That’s how it is... I think... that's life. “ (P5)*

This quote most vividly illustrates the main finding in this study. Participants who provide palliative care for their spouse are not grieving in a traditional manner; instead, they are “standing tall and fighting day after day until the end, no matter the cost”. To make this illustration more descriptive, we present what it means for caregivers to care for their dying spouses.

### **Bravely facing the challenges of “living with an illness”** by maintaining optimism, strong cohesion with their partner and a sense of joint destiny—***“We are fighting this together.”***

Caregivers are devoted to the role of a true-life partner. They are more or less aware of the severity of the situation but not willing to give up and mourn, while their loved one is still alive. They feel a sense of duty, reciprocity, love or devotion to their partner, and they want truly to *be with* him/her until the last moment. Based on their descriptions of their experience with challenges they faced while providing care, we concluded that they do not want to surrender or abandon their partner in this “battle” by showing weakness and negative emotions, instead they try to maintain positively spirited, brave, and supportive in every way. By analyzing the experiences of anticipatory grief in caregivers, we focused particularly on their thoughts and beliefs about the impending death of their spouses and about the care itself—the cognitive aspect of anticipatory grief. The analysis indicated that caregivers were most committed to providing support to a sick spouse, in a physical, psychological and spiritual sense, while trying to put their coping with the situation in the background. Respondents generally reported similar views presented in the themes below.

***“I do not want to think about it now.”*** – Avoidance or postponing the thought of coping with the death of a spouse.

The participants were generally reluctant to actively think about the death and dying of their spouses, nor to completely indulge in feelings in order to face the impending loss actively.

“(Sigh) What can I tell you. In a moment, yes, I’m thinking about death, and then I put that aside.” (P2)

***“I have to be brave!”*** – Maintaining optimism and hope by self-encouragement; understanding the inevitability of death, but having a hard time accepting it emotionally.

It was important for the respondents not to lose hope and optimism.

“*I try to think as positive as possible. Because, if you go into that negativity ... it eats you up.” Then you start to feel bad*.” (P5)

Therefore, the caregivers try to encourage themselves, their ill spouses and other family members.

“*Sometimes I think, God, it won’t happen so quickly, so maybe it will all be good. So, I look at the situation positively, I never think about it in terms that it will be over soon. Somehow I hope that it will all end up well, somehow (smile).”* (P1)

*“Listen, this is the end, he tells me, and then I tell him – come on, it will get better.”* (P1)

… *I saw him suffer, how hard it is for him. He tells me: why don’t I die? This is not life, this is suffering. I hug him and say: kitty, this shall pass too. You know it has already been so critical, so here we are again… so it will be good again.”* (P8)

“*I am preparing for the death of my spouse. I comfort my children every day so that it does not come as a shock to them, and I am preparing them. .. And most of all I must be brave in front of the children, so as not to panic…”* (P6)

***“I have to accept.”***—Rationalization for accepting death and dying as a normal part of life.

Some participants try to look positively at what they are experiencing; they hope for the better, but they are also realistic in anticipation of death. They encourage themselves and convince themselves of the naturalness of death.

“… *A person must understand this and move along… we all go there… there are no exceptions…”* (P8)

In the case of younger participants, who also take care of their spouse for a shorter time, it is evident that they do not yet accept the impending death. They are probably conscious of it, but they are still hoping for a cure. They also state that it is difficult for them to accept that the disease is changing the spouse for the worse.

“*I still hope that something will improve. When I look at the beginning, this is now as if I have a different person next to me, because he has changed his character… quite negatively. He does not accept it, it is difficult to deal with it, so we are going through a lot. He can be very unpleasant, and that is hard for me because once I had my husband, and now, after two months, he is a different person*.” (P7)

Some participants accept the impending loss and, in some way, try to face it. From their statements, it can be observed that they are trying to prepare for the imminent loss by encouraging themselves.

“*With faith and prayer, I think I will be able to get over it. That’s what I can say at first. And as for the other people, ..I just have to face it on my own, deal with it..*” (P7)

***“What else can be done?”*** – focusing on present/immediate future and current problems, as avoidance or postponement of the prospect of a loss.

The participants expressed that they do not give up or do not want to accept as long as there is something that can help.

“*I begged them, I will come wherever I need to go. I begged the doctors: I will do everything in my power, anything that I can do*… *Then I called and asked what was the next step? I know that the radiation is over, but the man is mobile, I understand that the man laid down in bed, that you can see that he is helpless, but that is not it. Then it hits you because... what’s the next step?”* (P5)

This optimism or “super strength*”* is not present all the time. Participants recall many (private) moments when they felt anxiety about the future but were able to “pull themselves together” for the sake of the partner (and/or children). For caregivers, this strength often comes from within, from other family members or God. One of the most important themes in this study was the role of spirituality – the participants reported often praying in difficult moments and relying on God’s plan. The following elements of spirituality in anticipatory grief were expressed:

***“It’s all in God’s hands.”—***Seeking meaning and comfort in reliance on God’s providence and will.

Based on data, searching for the meaning of life and death is essential to the respondents, as well as surrendering to God’s will and providence. In their answers, each of the respondents mentioned some of the religious practices they typically practice, stating that it is one of the most important ways for them to cope with a complex and challenging life situation, such as the impending death of a spouse. Spirituality helps the participants to cope with the situation and to accept it.

Participants’ responses describe practicing their faith in a way that they pray, talk to God, go to Mass, and read religious literature. This spiritual side was important to them when caring for their spouse, because they do not want to do anything to hurt or aggrieve their spouse and because this is in line with their beliefs.

“*Sometimes I panic, but I think of God again. God has given me discernment, I wouldn’t be able to discern it if it weren’t for God’s will and faith. I believe in God, so I take comfort in that. I look at the situation from God's side*.“ (P6)

The spirituality of the participants was visible in the answers where they tried to grasp the deeper meaning of what is difficult for them to accept.

“... *I don’t think a man is ever ready for that. It’s a flow that comes spontaneously; you may be aware of it, but deep inside you try to make it easier for yourself. Because then when it happens, it just happens ... Maybe that is the moment when you have to come to terms with it because otherwise, it cannot be. It is a sequence of life...*” (P5)

Informal caregivers were often in a state of disbelief and questioned the meaning of life and death. In search of meaning, the participants ask themselves in disbelief why this happened to them, thinking about how they had a beautiful life together and shared plans and a future, which they are now somehow losing. Questioning God's plans does not lead the respondents to concrete answers, and this makes some of them even sadder.

“*Why him; he is still relatively young, why it happened to him ... Those are the questions haunting me*...” (P5).

“… *You think, God, why right now… Just when you think that now you will enjoy life with that husband and this happens*…” (P1).

Some participants reflect on the meaning of life when facing the imminent death of their spouse.

“*It’s nice when a person lives his life nicely ... You should look forward to the little things, you should say thank you God for that. The point of life is to understand that the older you are, you need less material goods and more spiritual growth. Growing in another sense, a nice word, a warm touch, a warm look ... There is so much in life that a man can do, and we miss to do it, and here it is*.” (P5)

### Repressing own personal needs and feelings while carrying the burden of care—“*How can I complain when he’s the one dying?”*

The second prevailing theme was the caregiver’s lack of preparedness to talk about their own feelings and capacity for coping with this situation. For example, some of them were surprised that the interviewer asked them how they feel instead of how their spouse was doing. Maybe it is unusual for them to be in the spotlight, because of their permanent focus on the needs, emotions and suffering of a spouse. The participants put their own needs aside. Also, many other explanations could be plausible, such as deflection, repression, avoidance (or some other defense mechanism) of the unpleasant thought of death and separation from spouse or living after spouse’s death. In searching for the meaning that participants ascribe to the impending loss of a spouse, our special attention was directed to the emotional aspect of anticipatory grief. We have identified different emotions and emotional states that dominated their answers—feelings of sadness, fear/anxiety, anger and helplessness, and interpreted specific reasons/source that caregivers attached to them. These emotions do not represent themes in our analysis per se but serve as an additional resource for interpreting specific emotional states of the caregivers that could provide us with a deeper understanding of this *meaning*.

- ***Feelings of sadness and grief*** over a spouse’s (undeserved) suffering and illness and facing that there is no hope for recovery.

During the interviews, the participants repeatedly pointed out that they felt sadness and that they were very sad. In addition, they often cried when answering questions and describing those difficult moments or situations in the care of their spouses that make them feel saddest.

Analysis of the participants’ responses suggested that caregivers feel sad for several reasons. Sometimes they were not able to explain in more detail why specifically, but it can only be concluded that they are overwhelmed by such feelings.

Some cited the spouse’s suffering as a reason for their sadness.

*„I am sad… I still look with sadness (tears). I can’t come to terms with it… I mean I can come to terms with the fact that he doesn’t walk, but when I see him suffer, it’s hard for me… it’s hard for me to watch*.” (P7)

Others highlighted that they felt sad because their husband was ill at all.

“*What is the hardest thing for me to deal with… (sigh) it is the realization that the disease is actually there, that he is seriously ill, that is the worst thing for me*.” (P1)

Grief over the “fate” of the spouses was also cited as the reason for sadness since in the opinion of the respondents, the spouses did not “deserve” it.

„*(Tears)… ..It is very hard, I keep crying. All my life, she struggled, she worked a lot, she sewed for the children and for me, for the neighbors, we were subtenants. She always did everything, paid the bills, took care of everything with the landlord, I would give her money, then she took care of everything. She never complained about the children, the house*.” (P3)

The hope of a cure that was not possible also led to a feeling of sadness in the respondents.

“*The hardest thing for me was that psychological aspect. I think he will get better, he will get better, but there is no progress, not the progress that I can see*.” (P6)

***-Feelings of concern, fear and anxiety*** about the lack of competence to provide the necessary and immediate medical care; imagining a future without a husband and reconciling work, family, and caregivers’ duties.

When it comes to emotional reactions related to the care for the spouses and the thought of their death, this theme dominated their experiences. It was noticeable that fear and anxiety were feelings that occurred for several reasons. Based on the answers, we categorized the reasons into several groups.

*Fear of lack of competence to provide the necessary care*: participants are faced with care tasks for which they were not adequately prepared and trained. However, they feel that they must (and want) to provide the best care and attention to the spouse. Examples of such care were most often related to the provision of health care, such as the taking care of therapy, the provision of special nutrition or the care of, for example, a catheter etc.

“*I’m afraid, the entire burden has fallen on me. I have to take care of the medicines, how it will be, what it will be, it’s a big task for me*.” (P6)

Such fear was expressed mainly by participants who provided care and nursing to a spouse whose condition had suddenly deteriorated in a short time.

When talking about the fear of feeling insufficient competence to provide care, it is important to single out the *fear of lack of competence to provide immediate emergency care*, i.e. the participants’ fear of responsibility for the spouse’s life, i.e. that they will not be able to “save” their spouse’s life by themselves, if needed.

“*Here, we are talking about the throat, potential suffocation ... I have a fear of whether I will know what to do, at the moment when it really, if it happens. Will I know how to help, on my own ... if he starts to suffocate ... that feeling of helplessness ... those moments are extremely difficult*.” (P5)

*The fear of loneliness and living without a husband* was also one of the fears amplified by the participants’ inability to reconcile the demands of their work and family roles and responsibilities.

“… *The hardest thing for me is actually the idea that one day he will be gone… and that I’ll be alone… and then I think ... Jesus, what am I going to do… because I’m not the type of a person who likes loneliness; I’ve always been with someone, I’ve never lived alone, and so on*.” (S1)

*Fear for the future of young children* – was expressed by a young participant. Daily life demands may amplify the fears of the informal caregivers, presenting the additional burden.

“*I am worried about the future of the children, because it is difficult for me to provide high-quality care to my children and to my spouse. I cannot reconcile those two sides. Because children have their own demands; they are still young, and he has his own demands. If I take care of him then I am constantly locked in the house and if I take care of the children then I have to go out with them. To take them to school, to socialize, to sports, to kindergarten. It's all very difficult, very, very difficult for me (tears).*” (P7)

The participants expressed that they feel anxious about the demands of everyday life and the need to fulfil obligations that they have not had to perform on their own so far.

“*I am afraid…. Look the other day I needed to change the light bulb in the bathroom. I was afraid I would fall, and then who would take care of him. ... If I get dizzy, and I am so nervous, I will fall, and then – who will take care of him*?” (P4)

The anxiety they feel is heightened with the responsibility that comes with providing the care.

*“… So now when he came home with that, when he got a drain for that liquid, there I panicked. I didn't know how to deal with that, what to do with that. They never explained it to me in the hospital. I did not know how to do that. That was the hardest thing for me so far.*” (P6)

The participants also described the helplessness they feel because of that responsibility for someone’s life.

“*I am afraid… what will happen, how will all that turn out. … as all the burden has fallen on me.*” (P6).

***Feelings of anger and helplessness*** aroused by the lack of better palliative institutional support for their spouse.

The participants’ anger also occurs when, in the absence of information, due to limited treatment options or insufficiently fast health care system, the spouses do not receive adequate care or information.

“*I will do everything in my power, but I want to know what will happen in maybe a month. We need to be informed*.” (P5)

Anger was also expressed over the lack of better support for the patients in need of palliative care, including the need for improvement of the national healthcare system, especially in the context of the COVID-19 pandemic.

“*I think that palliative patients are totally deprived. I don’t think there is enough understanding from health workers. I don't know, for example ... we have the current situation: my husband should have gone to the aquatic physical therapy; he is an immobile patient, and that is their first problem – they cannot ensure support for such a patient. Another problem is his pressure ulcers because of which he cannot go because there is no one to treat them. He was even told in one such aquatic physical therapy institution that he could come, but that he needed to be accompanied by a spouse who would take care of him. His answer was – but we have two small children, who will take care of those small children. Unfortunately, they said, we can’t help you. So, he needs rehabilitation urgently, and he can't get it because he is immobile and has pressure ulcers… and how are we going to solve that? He was sentenced to bed at the age of 39. He does not have the right to physical therapy 5 times a week, I don't know for what reason… and he is a young man*.” (P7)

*“.. as we all know, a specific situation like COVID slowed down the entire health system and everything was oriented towards COVID, but neglected the other side. That's the other side, that people suffer from other diseases, serious diseases, that's the time that health workers, doctors themselves note that they need to pay attention, orient themselves to such patients, COVID is not the only problem, that's how it is at the moment...”* (P5)

The participants’ anger was sometimes directed at themselves as well, and there was a prominent feeling of helplessness.

“*I’m angry because I'm not doing my job well, because I can’t dedicate myself to him and the children. And then there is my regular job, because I have to work and I have to earn money for the family. And I feel like I’m not good in any of that. It’s hard, all that at the same time… (tears)*”(P7)

Furthermore, our analysis showed that participants also feel anger and disappointment over their inability to receive adequate medical treatment or support.

“*What may hurt me the most is that you may find yourself in a situation where someone [medical staff] is giving up on you*… *It's disappointing when even though there is no help, I understand that death cannot be avoided, but it's also painful...when I got the letter...I'm not a doctor...but I understood...they gave up on him.”* (P5)

***“As long I can go on, I will push.”*** It has emerged in our analysis that such an active commitment to informal caring for the spouses, without adequate expression of their emotions, could lead to adverse outcomes. Such as impaired health of the caregivers that includes exposure to stress associated with the caregiver’s burden.

“*I also have health problems with my heart. I am undergoing tests right now because I experienced shocks due to this care for him. At night I stay awake, and I listen if he is breathing. When he was coughing, he had a bout of that cough; he could not catch his breath. That was all shocking to me. I got sick too, so I ended up in the hospital because I ended up being so weak*.” (P8)

The participants state that they are so preoccupied with providing care to their spouse that they neglect their own needs, and they also neglect the health problems they themselves have. They are physically exhausted.

*“I give everything for him. I give everything to him. I pray to the dear God to give him strength and let him give me the strength. I pray not to make a mistake, not to defile my soul with sin. If he asks or wants something or criticizes me, I can’t just leave him… I won't… because I'm afraid. My colleagues tell me: come on, let him go, go out, leave him. I say: my dear girls, I'm afraid of dear God. I don’t want to do that; I do not want to leave, for him to be left alone for 5 minutes. When he goes to sleep, I go out a bit. This is my life now – I go wherever he goes. He walks on crutches to the fence outside, a little to the street, he looks around. I'm there, and I follow him because he is weak. We bought everything we possibly could buy to strengthen his body. I say to everyone: I'm not the only one who has a patient at home.“* (P8)

The participants state that they do not even think about a break.

“*You know, at night I need to face him, and I don’t sleep. I jump right away when he starts moving down. Because I am afraid he will fall*.” (P2)

According to the informal caregivers of the spouses, other people also notice their burden and encourage them to take more care of themselves. All aspects of such exhausting and demanding care and the position of a caregiver are best described in this statement:

“*This is my task now, and I feel a duty. I put everything into him. But I have to tell you, at one point, I felt completely empty”, as if everything came out of me. I was so weak…, I gave all my energy. I became so weak, and the children saw that and said: mom, we have to do something, do something, you can't fall too; if you fall, what are we going to do with dad. He only trusts you. I said: just give me some time…, I'll be back*.” (P8)

### *“*Living in the present” by taking an active role in providing care for the ill spouse /and family – positive and proactive approach in organizing activities of everyday life by living day-by-day - *“I am surviving.”*

This thought captures their sense of surviving one day at a time, living in the moment, not knowing what tomorrow will bring, not planning the future or simply not thinking about it. On the one hand, this state probably helps them stay dedicated to problem-solving—in tasks of care, and on the other hand, delays dealing with the emotional burden of impending loss of the spouse. Thus, caregivers are not (so much /or all the time) focused on the matter of living, dying, or living after the death of the spouse. Instead, they are more focused on emerging everyday problems that come with care, such as taking care of decubitus, catheters, resolving pain, handling other household chores, taking care of children, etc. Surviving, in this sense, means providing care for partner and family by organizing everyday activities, with minimum introspection or reflection on personal strengths, needs, plans or even taking joy in living.

Three themes emerged in our analysis that pointed out several strategies that participants used to deal with the challenges of care on a daily basis. These strategies describe the *meaning* of living in the present, or in other words, what do caregivers do to survive these challenges? This could be interpreted as behavioral aspect of anticipatory grief.

One theme is *self-motivation*. Caregivers maintain a positive self-approach in coping with the challenges of providing palliative care for their spouse (self-encouragement, self-reliance, self-efficacy, acceptance of the caregiver role). The second theme is *seeking support* from medical experts, friends, family, God and their ill spouses. Participants indicated the need for emotional, physical, spiritual and support regarding gaining professional medical advice, instructions and help. The third theme describes their commitment to take an active role in providing care.

#### Self-motivating

In order to alleviate the situation in which the participants found themselves, they try to focus on the present. At the same time, they are optimistic and try to view the situation optimistically. In several ways, they are trying to focus on their own strengths and resources.

#### Self-encouragement

They try not to give up and try to strengthen themselves by looking at the situation where they find themselves as positively as possible.

“*I try not to think in the long run, but from today to tomorrow ... I try to think as positively as possible, because if you start wallowing in negativity... it will eat you up. Then you start to feel bad*.” (P5)

The participants are encouraged with their previously acquired caregiving experience.

*“It’s not hard and difficult for me so far because I went through the same thing with my mother before… it will be seven years without her now. She also had colon cancer and I was the only one taking care of her, so I have a little experience. I can't say it's hard for me.“* (P1)

#### Self-reliance and self-efficacy

Multiple participants felt it was easier for them due to the earlier loss of a loved one.

“… *Because my mother died 18 years ago, and thus I faced the death of a loved one*…” (P7)

It is important for them to rely on their own strengths to protect others.

“*I told myself: look, you have to, he only has you. I mean, he has a brother, but a brother has a family. There is no one else to take him to the toilet, who will do the washing, and everything else that goes with this…*“ (P4)

#### Acceptance of a caregiver role

It was common for all respondents that they accept the role of carer as their duty and obligation.

“*I didn't have the strength, but I somehow got used to it*." (P3)

“*Listen, he has been sick for a long time. I'm with him all the time. I accept everything. How can I put this – I do not find this difficult. I accept it as my obligation. I don't know if you can understand; as the disease comes, I accept that*.” (P2)

Some felt the care of the spouses was their own duty and as an opportunity to repay them because they once cared for them.

“*When I had a fever, she nursed me and jumped around me, I can never forget that. Then I tell her: you were helping me before, and now I help you. So she somehow accepted it now*.” (P3)

Some participants mentioned other strategies that help them with care. The participants try to maintain other aspects of their own lives, such as taking care of the household, pursuing hobbies etc.

“*I like to read, that saves me. I like to read everything, books about cooking, etc. And you know what else I love, medicinal herbs*... “(P2)

Some of the participants find solace in participation in family activities.

"I play with my grandson; when I went on a trip with them, it helped me." (P3).

Also, by reading the literature on self-help, the respondents try to help themselves.

“*I read a self-help book, I read a lot. My daughter got it all for me. She said: mom, you need to read to get stronger. And I did get stronger*." (P8)

#### Seeking support

Health-related requirements of the spouse's condition present the most difficulties in the daily functioning of all the participants. Due to such a health condition and the complexity of care, they expect the most support from experts, both regarding the medical procedures, but also emotional support.

“*I was comforted the most by the nurse and doctor from the palliative team. The first time they came, I was afraid; there were medicines to do, and other things, I was afraid that I would make mistakes. They said you will not do anything wrong; you have our support. I didn't even know that I had to call those who will drive my spouse somewhere, the ambulance. I knew none of that; I never encountered any of that. But they come to me, and they give me advice, and that helps me. They give me good advice. Some people also gave me poor advice. So, it is important to me that I can rely on healthcare workers to always call them and ask. I am afraid, as all the burden has fallen on me.*” (P6)

The participants stated that they were in great need of specific medical help and health care services for which they are not sufficiently educated.

“*I need help with wound dressing, pressure ulcers, because I don't like those things. I've never been interested in medicine, or wounds, or anything, and then when I had to do it for the first time, the community nurse helped me. But, nevertheless, when he needs it, I have to deal with it, with that wound and with the blood and everything. That is very difficult for me*.” (P7)

Several respondents do not know how to deal with the very moment of death and what it will look like, and they expect to be supported even in such a situation.

“I will need support if the death comes. I mean, you never know how that will be." (P1).

The support from family and friends was considered important by the participants. They said that friends and family were people they could always rely on, talk to, and make it easier for themselves. It is important to them that family and friends support them when they are worried and lonely.

“*I’m calling my friend, I'm calling everyone around and a neighbor... I’m looking for someone, I'm asking for another person's help*.” (P1)

“*I have two friends; they are like sisters to me. They were there for me for whatever I needed – financially, as humans, with love, for everything.”* (P4)

The participants find support from family and friends essential and valuable in situations when they cannot independently perform all the requirements in care, communication with a physician, etc.

“… My son sends test results via email, I don’t know how to do that…” (P6).

An ill spouse is also a source of support for informal caregivers. Spouses support each other, as in other experiences throughout their lifetime together.

“*He was always my consolation. Then I calm down, so I don't go crazy (laughs). You know, we're both cheerful; we laugh at our suffering. He is a very cheerful person by nature, and as long I can go on, I will push*.” (P2)

“*We do it alone, we do it all together. We cry together. She was a consolation to me; I was always the emotional one*.” (S3)

The participants indicated that they ask for God’s help and support. Such spiritual support helps them to continue caring for their spouse.

“*I constantly pray the rosary; I pray to St. Anthony and St. Joseph. Whenever it was hard for me, I started praying, and it helped me. So prayer and "Someone" helped me. I want to tell you that I'm saving myself with this. Whenever I was afraid, when I was most afraid of what would happen, when I had no solution, I would start praying out of fear. I mean, I usually pray, and when I don’t know what to do, I ask God to help me*.” (P2)

“*I say, with faith, I helped myself very, very much*.” (P8)

#### Taking an active role in providing care

As the disease progresses and the care becomes more demanding, the participants state that they are more actively engaged in planning and implementing care.

“*I take care of my husband from 0 to 24 hours. I do everything related to bathing, changing diapers, urinary bags, nursing pressure sores, feeding. He eats on his own, but I need to prepare a meal. I do absolutely everything: turning in bed, moving to a wheelchair, and so on. I do absolutely everything*.” (P7)

Spousal care includes hospital visits, application of therapy, maintenance of hygiene and care, and provision of emotional support to the spouse. In addition, they try to help further and do everything possible for the spouse.

“*He went to the hospital, and it was as if he had fallen from the sky. He doesn't know where he is. I wrote to him, and my son told the nurse – look, my mother wrote him a letter.”* (P2)

The participants independently seek information about illness and care for their spouses.

“*I read an awful lot on my own. When I came across this disease, four and a half years ago, I didn't know anything about it*." (P8)

## Discussion

Results of this study pointed to three core meanings caregivers ascribe to the experience of the palliative care providing and the impending loss of a spouse.

***“We are fighting this together.” ***Caregivers bravely face the challenges of “living with an illness” by maintaining optimism, strong cohesion with their partner and a sense of joint destiny.

***“How can I complain when he’s the one dying?”*** Caregivers tend to repress their own personal needs and feelings while carrying the burden of care.

***“I am surviving.”*** Caregivers tend to “stay positive” and focus on “living in the present” by taking an active role in providing care for the ill spouse /and family.

The participants expressed that they feel sadness, grief, worry, fear, anxiety, anger, and helplessness. They try to postpone the thought of death without losing hope and optimism. They encourage themselves, convince themselves of the naturalness of death, and focus on the present and current problems while not accepting the prospect of a loss. They try to find meaning and comfort in leaving the situation to God’s providence and will. They are in disbelief and question the meaning of life and death. In coping with these reactions, they seek support, work on themselves, and actively dedicate themselves to caring for their spouse, even with negative consequences for their own health.

These three broad meanings show the true complexity and ambivalence of caregivers’ emotional and mental state. They were torn between their *true* feelings and the feelings they thought they *must have*. This assumption is not true for all caregivers; individual differences in coping strategies between caregivers and their spouses were also evident. They had different reactions to accepting the impending death of a spouse.

This finding was expected and that was the reason why we used the idiographic approach to investigate particular and unique details of each case. The interpretative phenomenology allowed us to describe and deeply understand what it means to provide palliative care for the spouse and how caregivers cope with the challenges of everyday life. Analysis, which included our reflections, provided us with many additional insights. Our participants differed in their coping strategies, quality of relationship with their spouse, acceptance of the imminent death of a spouse, etc. As an example, we noticed that older participants, who provided care for their ill spouse for a longer time period, who had the experience of the previous death of someone close to them (e.g. mother, son, etc.), revealed the different experiences of care and acceptance of the caregiver role than the younger participant who provides care only a few months. The latter still had a problem with accepting diagnosis and managing tasks of providing care for spouse along with upbringing small children and working a daily job. In future research, these differences should be further investigated with different methods of analysis—that includes case comparisons on a larger purposive sample.

These results aligned with the description of anticipatory grief from previous research. Described experiences included a range of intensified emotional responses mentioned in the literature, such as separation anxiety, existential aloneness, denial, sadness, anger, resentment, guilt, exhaustion, and desperation [15]. In addition, a heightened awareness of mortality, and inability to plan for the future, were also found in previous research of anticipatory grief among spouses of patients with cancer [16].

Previous research also pointed out the presence of emotional and cognitive ambivalence between recognition and acceptance of loss, along with the need to protect oneself from this comprehension by sustaining hope –to keep functioning and continue providing care [17].

The recent systematic review and qualitative meta-synthesis of managing anticipatory grief in family and partners also showed that participants’ realization of imminent death gave them intense motivation (sense of duty) to provide care for their loved ones. They expressed that the need to protect, help, and support their loved ones motivated them to engage in caregiving and gave them the strength to get through the illness [18].

The study results showed that the anticipatory grief of informal caregivers manifested in emotional, cognitive, spiritual and behavioral reactions to the impending loss of a spouse. These reactions facilitate our understanding of anticipatory grief as a multidimensional phenomenon and are not theory-driven nor an attempt to validate any model. Its purpose is to present the variety of caregivers’ experiences of providing palliative care with an emphasis on what they feel, think and how they manage it.

The contribution of this study are aspects of the participants’ behavioral grief and how the grief is handled, confirming prior results. The results of this study are in line with similar studies conducted in other countries. For example, a study by Breen et al., conducted in Australia among informal caregivers who provide palliative care to family members or friends, concluded that emotional reactions show that caregivers are constantly fluctuating between indulging in grief and focusing on caring for a spouse [19]. Their feelings of thinking and beliefs are certainly influenced by the condition of the spouses, where they emotionally try to be encouraged and persevere in care. They are most preoccupied with providing care, letting go of mourning, trying to postpone thinking about it, stepping aside because it seems easier for them when they are not thinking, maybe thinking about losing them is too painful, or there are some other reasons.

In preparation for death, it is already known that even when caregivers are cognitively prepared, they are never fully emotionally ready for that challenge [19].

The informal caregivers’ grieving reactions point to processes that develop through different stages, with some caregivers in denial, some in depression, and some slowly accepting the likelihood of losing a spouse. This could be considered consistent with the grieving process described by Kübler-Ross, who described in 1969 that dying persons go through five stages of grieving, including denial, anger, bargaining, depression, and acceptance [20]. Kübler-Ross herself extended the application of those stages of dying to the situation of (anticipatorily) bereaved people in her 1969 book [20]. However, it needs to be highlighted that there is an opposition to the stage theory proposed by Kübler-Ross. For example, Wortman and Silver argued that the five stages were widely adopted by the healthcare professionals without solid evidence, which may lead to negative consequences for the bereaved, who are expected to go through the pre-defined stages. They highlighted that the course of grieving might be heterogeneous among many bereaved persons [21]. The review of Stroebe et al. summarized the criticism regarding the stage theory as follows: lack of theoretical depth/explanation, conceptual confusion and misrepresentation of grief and grieving, lack of empirical evidence, the availability of alternative models, and potentially devastating consequences such as undue expectations about the course the grief should take [22].

Anger directed at what is happening to the informal caregivers is also manifested in their cognitive and spiritual questioning and searching for the meaning of what is happening to them.

It is already known from the experiences of Viktor E. Frankl, a famous neurologist and psychiatrist who shared his experiences as a concentration camp prisoner, that in difficult situations, the search for meaning helps individuals cope with difficult situations [23]. Therefore, giving meaning is very important to caregivers to help them persevere in caring.

A number of participants in our study stated that spirituality is important to them. Through spirituality, they find strength, comfort and encouragement to continue caring, believe that their commitment and care are focused on obedience to God's will and thus make it easier for themselves. In 2021, Benites et al. published a systematic review about the experience of spirituality in family caregivers of adult and elderly cancer patients receiving palliative care. They found that family caregivers express their spirituality multidimensionally, giving meaning to the care they provide, and reassessing the meanings of their lives and their suffering. The authors concluded that investigations of suffering and spiritual needs of family caregivers in this context might be valuable to inform comprehensive and multi-professional psychosocial care [24].

Caregivers’ emotional reactions indicate that they are trying to maintain all aspects of life while cultivating hope and optimism; they focus their energy on active care and that in some way, they cannot or do not want to face the loss. According to the Kübler-Ross model, it is known that denial is also one of the phases of mourning [20].

Those with a long experience of care may have managed to "go through" different stages of mourning according to Kübler-Ross [20], i.e. they are calm, they accept their destiny and justify it by God's will, the laws of nature, the desire to end the suffering of their partners, *et cetera*. The spouse’s acceptance of imminent death undoubtedly plays a role in this dynamic. On the contrary, those who recently discovered the spouse’s life-threatening diagnosis suffered from denial and anger.

Their excessive and constant focus on providing care may be why they cannot accept the loss, which is consistent with earlier results [25]. The results of Toyama et al. indicated that these family caregivers had two primary roles, whereas one role is a family member and another role is a caregiver. The family caregivers felt trapped in their caregiver role. The narrative approach helped them to make the transition into the role needed for coping with the loss [25]. Hopefully, conversations held with participants in this study also helped them cope with the imminent loss.

It is known that not all persons are equally prone to developing higher levels of anticipatory grief and that this grief can have varying degrees. Furthermore, anticipatory grief encompasses many areas of an individual’s life – including intrapsychic elements (e.g., emotions, cognition, planning), interactional elements (i.e., relating to and helping the dying patient), and family and social elements (i.e., relating to family and others) [26].

These processes can help grieving people predict loss and continue their involvement with a dying patient. However, for some individuals, this experience can be psychologically exhausting. Many researchers have described how anticipatory grieving is related to various factors, including the background characteristics of the caregiver and the dying patient and his / her illness, psychological distress and the harmful consequences of coping with the loss [15, 27, 28].

Because anticipatory grief is associated with various outcomes, risk factors for this condition deserve more detailed consideration. Burke et al. published a study in 2015 in which they investigated in more detail the risk factors associated with anticipatory grief. They included 57 family members of terminally ill veterans entitled to hospice and receive palliative care services. Researchers assessed participants’ psychosocial factors and conditions. Higher levels of anticipatory grief were found in families characterized by relational dependence, lower education, and poor grief-specific support, who also experienced discomfort with closeness and intimacy, neuroticism, spiritual crisis, and inability to comprehend the loss. Burke et al. conclude that, based on the findings of their sample, anticipatory grief appears to be part of a group of factors and related ailments that require early monitoring and possible intervention [29]. Caregivers in our sample differed based on the sex, age and duration of care for their spouse. In addition, they described their experiences in different ways. However, we did not analyze their characteristics and risk factors in more depth.

What helps caregivers the most in dealing with the current situation is seeking support from health professionals in palliative care; they mostly need explanation and communication that confirms that they take good care of their spouse [30]. However, the captive role of caregivers cannot be viewed in isolation from the broader social environment in which the role of informal caregivers is not officially recognized and particularly in the setting of underdeveloped palliative care in Croatia [31].

According to Lazarus and Folkman’s transactional theory of stress and coping [32], the need for coping arises in intensely emotional environments. In the case of this study, that translates to expecting the inevitable and imminent death of a spouse. In this severely stressful environment, caregivers are faced with stressors appraised as uncontrollable, so they turn to strategies that aim to down-regulate negative emotions.

In our study, participants revealed several helpful emotion-focused coping strategies. These strategies were a distraction (cognitive avoidance of dealing with the death), self-encouragement (e.g. looking for the positive in the current situation), seeking emotional support or allocation of problem-solving to a third party (e.g. making sense in seeking God's support) and acceptance of *the faith* and current caregiver role (redefining situation and their position in it). Caregivers that provided care for a more extended period or/and caregivers that were older (lived for a longer time with a spouse) maybe have had more time to adjust to the thought of loss and were more often using acceptance as a coping strategy. On the other hand, younger caregivers, or caregivers that recently found out the prognosis of spouse's illness, were more often using distraction as a coping mechanism. This insight was also consistent with the phases of the grieving process described by Kübler-Ross [20]. Problem-focused strategies that caregivers used in this study were seeking social (and professional) support regarding performing care to their spouse or organizing care plans. Also, they used techniques for self-motivation and self-help.

Our findings of adverse effects of informal caregiving confirm prior research results. Lennaerts-Kats et al. reported that palliative care presents a very demanding experience for family caregivers. Such caregivers can have psychological problems long after the person they nursed has died. There is a need to increase the awareness of health professionals about the needs of families and grieving caregivers, which can mitigate these long-term adverse effects [33].

Thomas et al. recommended that the quality of clinical practice could be improved if specialist palliative care teams in the community context recognized and responded to the significant support needs associated with relevant background concerns of family caregivers [34]. It is also worth emphasizing that it is currently unknown which interventions could help to alleviate anticipatory grief [35].

During our interviews, it was observed that participants were grateful for the opportunity to share their experiences with someone from the health care field. In addition, all the participants were very collaborative and openly shared their feelings and thoughts with the interviewer. To our best knowledge, this is the first study of this kind in Croatia. Considering the specifics of palliative care organization in Croatia, it is worth emphasizing that similar topics emerged as in studies conducted in countries with higher income.

A potential study limitation could be the previous inexperience of the first author in conducting qualitative interviews. However, the first author was trained by an experienced team. Furthermore, the sampling was purposeful, and thus the findings of this study cannot be generalized. Also, in this study, we included only one man, and we did not have balanced characteristics of the sample in terms of the participants’ characteristics, such as their age and employment status. Including more diverse participants could have brought different perspectives to the study.

Future studies could include a quantitative study design and the needs of informal caregivers who provide palliative care to their spouses in Croatia on a large sample. Furthermore, as a prerequisite for studies on anticipatory grief, the Anticipatory Grief Scale (AGS) [36] should be validated for the Croatian language and population. Also, in future studies, it would be interesting to consider the stage of the disease and the perception of the imminence of death because these variables can affect the caregivers’ emotional, cognitive, spiritual and behavioral responses. Finally, future research should also focus on other family caregivers beyond spouses.

In conclusion, a qualitative study conducted in Croatia showed that anticipatory grief presents emotional, cognitive, and spiritual challenges to spouse caregivers in palliative care. The contribution of this study was to gain a deeper understanding of the experience of care providing for the ill spouse and the experience of grief or how the grief is handled, confirming prior results. The experiences are generally similar to all caregivers, but the support and help they need from palliative care require improvement.

## Supplementary Information

**Additional file 1:** **Appendix 1.** Written information for participants about the study. **Appendix 2.** Informed consent. **Appendix 3.** Questions for the semi-structured interview.

## Data Availability

Transcripts from the interviews, conducted in the Croatian language, are available from the corresponding author on request.
