# Supplementary material for: Anticipatory grief and experience of providing at-home palliative care among informal caregivers of spouses in Croatia: a qualitative study
Source: BMC Palliat Care. 2022 Nov 18;21:199. doi: 10.1186/s12904-022-01093-1 (PMC9672539; doi:10.1186/s12904-022-01093-1)
Supplement: Supplementary file 1 — Additional file 1: Appendix 1. Written information for participants about the study. Appendix 2. Informed consent. Appendix 3. Questions for the semi-structured interview. [file 12904_2022_1093_MOESM1_ESM.docx]

**Appendix 1: Written information for participants about the study**

Dear Sir/Madam,

we invite you to participate in a study on the grievances of informal caregivers providing at-home palliative care to their spouses. The study is conducted via face-to-face interviews in your home or at another location that you suggest, which will ensure the privacy of the conversation. If you agree to participate in the study, you will arrange an appointment with the nurse at a time that suits you. The interview will last a maximum of one hour. In the interview, you will be asked pre-defined questions about your care for your spouse, who is in palliative care. In addition, you will have the opportunity to provide any additional information that you think is important and may not have been covered by pre-determined questions.

I would kindly ask you to participate in the study because your experiences are very important for improving the future support for informal carers in palliative care. Please answer all questions spontaneously and honestly.

The study protocol was approved by the Ethics Committee of the Catholic University of Croatia and the Ethics Committee of the Health Center Zagreb-East.

There are no risks associated with this research, i.e. the level of discomfort you may experience by participating in this study is not higher than the one you experience in everyday life situations. Participation in the survey is anonymous. The collected data will be used only for the subject research and will be stored in digital form on the computer of the research administrator, password protected.

Only researchers will have access to the data. Only the person interviewing you will know your identity. Your details will then be anonymized, so other team members and third parties will not know who participated in the survey. In further processing, only anonymous results will be used. The results will be used for a diploma thesis, research articles and lectures.

Participation in the research is voluntary and based on your consent, and you have the right to withdraw from participation at any time without any consequences.

Under applicable law, you have the right to access your personal data, the right to ask for correction, deletion, restriction of the processing and portability of personal data, and the right to object to the processing and file a complaint with the Personal Data Protection Agency.

If you want to be informed about the results and conclusions of the study, or you have questions or requests regarding the study, don't hesitate to get in touch with the principal investigator at the e-mail address (Prof. Dr. Livia Puljak, livia.puljak@unicath.hr). Furthermore, if you have any complaints about the study or are concerned about something you experienced during the study, please get in touch with the principal investigator who oversees the implementation of this research. (Prof. Livia Puljak, PhD, livia.puljak@unicath.hr).

We kindly ask you to respond to this invitation and to participate in the study.

Kind regards,

Jelena Bilić, Registered Nurse

**Appendix 2. Informed consent**

INFORMED CONSENT

1. I certify that on the date ________ in the place ____________ I have read this notice for the above described scientific research and have had the opportunity to ask questions.

2. I understand that my participation is voluntary, and I may withdraw at any time, without giving reasons and without any consequences, health-related or legal.

3. I understand that my statements will be accessible to responsible individuals, i.e. the principal investigator and other researchers in the team, members of the Ethics Committee of the institution (Health Center Zagreb-East) where the research is conducted and members of the Ethics Committee of the University (Croatian Catholic University) that approved this study. I give permission to these individuals to access the data I will provide in this research.

4. I accept/do not accept that my family member is familiar with my participation in this study.

5. I would like to participate in this research.

**Acceptance to participate**

I was informed about risks and benefits of this study. Thus, I confirm with my signature that I **ACCEPT** to give my permission for participation in this study:

**Participant**

Signature: ______________________________________

First name and surname (block letters):_______________________________________

Date: __________________________________________

The person in charge of informing the participant and obtaining informed consent for participation in the study:

**Researcher**

Signature: ______________________________________

First name and surname (block letters):_______________________________________

Date: __________________________________________

**Refusal to participate**

I was informed about risks and benefits of this study. Thus, I confirm with my signature that I **REFUSE** to give my permission for participation in this study:

Signature: ______________________________________

First name and surname (block letters):_______________________________________

Date: __________________________________________

**Appendix 3. Questions for the semi-structured interview**

Introductory note:

Hello, I am Jelena Bilić, a nurse and a graduate student at the Catholic University of Croatia (HKS). I am conducting research to write a diploma thesis at HKS. The study aims to gain insight into the experiences of caregivers of spouses receiving at-home palliative care. Therefore, I kindly invite you to participate in the study because your experiences are very important for improving future support for informal carers of people needing palliative care.

For the purpose of the study, I would like to interview you and ask some questions. The conversation would be recorded and later used to write research results. I would use all the data anonymously, and you can withdraw from participating in the study at any time. Would you please confirm to me if you understand and whether I have your consent to participate and record the interview?

*(After the respondents sign the informed consent, the questions are asked)*

1. Can you briefly introduce yourself and say something about yourself? (How old are you? What is your occupation? What level of education did you complete?)

2. Can you please describe who you care for and how?

3. How long have you been caring for ……. (Person's name)?

4. Can you tell me a little more about what it is like to care for a spouse (name) who is suffering from an incurable disease?

5. How have you viewed his / her condition so far?

6. How did you feel when you found out the spouse was suffering from an incurable disease? (sub-question: Have feelings changed over time?)

7. When you think about it, what is the most difficult for you at the moment? (sub-question: What worries you the most? What do you find most challenging to deal with? Have you had this feeling before, or has that feeling changed? What has been the most significant barrier for you as a caregiver so far?)

8. What usually helps you when you feel that way?

9. What is your opinion about the need to prepare for the death of a loved one?

10. Based on your life experience so far, who do you think might help you? What would you say you need?

Thank you for participating in this study
